# Pioglitazone and risk of bladder cancer among diabetic patients in France: a population-based cohort study

**DOI:** 10.1007/s00125-012-2538-9

**Published:** 2012-03-31

**Authors:** A. Neumann, A. Weill, P. Ricordeau, J. P. Fagot, F. Alla, H. Allemand

**Affiliations:** Caisse Nationale de l’Assurance Maladie, 50 Avenue du Pr André Lemierre, 75986 Paris Cedex 20, France

**Keywords:** Adverse effects, Bladder cancer, Cohort, France, Pharmacoepidemiology, Pioglitazone, Reimbursement database

## Abstract

**Aims/hypothesis:**

Previous studies have suggested an increased risk of bladder cancer with pioglitazone exposure. We aimed to investigate the association between pioglitazone exposure and bladder cancer in France.

**Methods:**

This cohort study involved use of data from the French national health insurance information system (Système National d'Information Inter-régimes de l'Assurance Maladie; SNIIRAM) linked with the French hospital discharge database (Programme de Médicalisation des Systèmes d'Information; PMSI). The cohort included patients aged 40 to 79 years who filled a prescription for a glucose-lowering drug in 2006. The cohort was followed for up to 42 months. Pioglitazone exposure was modelled as a time-dependent variable and defined by having filled at least two prescriptions over a 6-month period. Incident cases of bladder cancer were identified by a discharge diagnosis of bladder cancer combined with specific aggressive treatment. Statistical analyses involved a multivariate Cox model adjusted for age, sex and exposure to other glucose-lowering drugs.

**Results:**

The cohort included 1,491,060 diabetic patients, 155,535 of whom were exposed to pioglitazone. We found 175 cases of bladder cancer among exposed patients and 1,841 among non-exposed patients. Incidence rates were 49.4 and 42.8 per 100,000 person-years, respectively. Pioglitazone exposure was significantly associated with bladder cancer incidence (adjusted HR 1.22 [95% CI 1.05, 1.43]). We observed a dose–effect relationship, with a significantly increased risk for high cumulative doses (≥28,000 mg, adjusted HR 1.75 [95% CI 1.22, 2.50]) and long duration of exposure (≥24 months, adjusted HR 1.36 [1.04, 1.79]).

**Conclusions/interpretation:**

In this cohort of diabetic patients from France, pioglitazone exposure was significantly associated with increased risk of bladder cancer.

## Introduction

Pioglitazone is an oral hypoglycaemic agent that has been marketed in the USA since 1999 and in Europe since 2000 (France since 2002). According to the European public assessment report for pioglitazone [1], pre-clinical studies in rats have shown an association between pioglitazone exposure and bladder tumours. However, pioglitazone was not genotoxic or carcinogenic in mice, and tumours were observed only in male rats. A possible biological association exists, with a potential mechanism linked to the ‘predisposing’ capacity of peroxisome proliferator-activated receptor (PPAR) and/or PPARα agonists for bladder tumours [2, 3].

A randomised clinical trial (PROspective pioglitAzone Clinical Trial In macroVascular Events; the PROactive study) evaluated pioglitazone with a mean follow-up of 34.5 months [4, 5]. Fourteen cases of bladder cancer were observed in the pioglitazone group (0.5%) vs six in the placebo group (0.2%). After a blinded review with external experts, bladder cancer was diagnosed during the second year of exposure in six cases in the pioglitazone group vs three in the placebo group [4, 5].

A cohort study of the Kaiser Permanente Northern California (KPNC) database [6] included 193,099 patients (30,173 patients exposed to pioglitazone and 162,926 not exposed). The median time from first prescription for pioglitazone to the end of follow-up was 3.3 years. The de novo bladder cancer incidence rate was 81.5 per 100,000 person-years for patients exposed to pioglitazone vs 68.8 per 100,000 person-years for patients not exposed. After adjusting for age, sex and use of other glucose-lowering drugs, the association between pioglitazone use and bladder cancer risk was not significant (HR 1.2 [95% CI 0.9, 1.5]). Another analysis of a similar cohort found no association between pioglitazone exposure and cancer in several other sites [7]. However, the study revealed a significantly increased risk with exposure duration >24 months (fully adjusted HR 1.4 [95% CI 1.03, 2.0]). Long-term follow-up of this cohort is ongoing.

On the basis of these findings, the US Food and Drug Administration (FDA) issued a safety announcement in 2010 informing healthcare professionals and patients about its ongoing safety review but did not conclude that pioglitazone increases the risk of bladder cancer [8].

On the initiative of the French medicines agency (Agence Française de Sécurité Sanitaire des Produits de Santé; AFSSAPS), we conducted this study to investigate a possible association between pioglitazone use and bladder cancer incidence in a historical cohort of patients with diabetes mellitus in France. We used a methodology similar to that used in the KPNC study but with a much larger population. To assess the specificity of a possible increased risk of bladder cancer associated with pioglitazone exposure, we also investigated the association between pioglitazone exposure and cancer in other sites.

The results of this pioglitazone study were presented to the AFSSAPS, which decided to suspend the use of pioglitazone in France in June 2011 [9].

## Methods

### Data source: SNIIRAM and PMSI databases

The French national health insurance system consists of several specific schemes that cover the entire population (64.7 million inhabitants in 2010). The general scheme covers all French employees and represents about 75% of the population. The national health insurance information system (Système National d'Information Inter-régimes de l'Assurance Maladie; SNIIRAM) contains individualised, anonymous and exhaustive data on all reimbursements for patient health expenditure [10], including medicinal products as well as outpatient medical and nursing care, prescribed or performed by healthcare professionals. The SNIIRAM database does not provide any direct information about the medical indication for each reimbursement but does contain the patient's status with respect to full reimbursement of care related to a severe and costly long-term disease (LTD), namely the LTD diagnosis encoded in the International Classification of Diseases, 10th edition (ICD-10; www.who.int/classifications/icd/en/). In the general scheme of the French national health insurance system, nearly one in seven patients received full reimbursement for an LTD in 2008 [11].

Information from this database can be linked to the French hospital discharge database (Programme de Médicalisation des Systèmes d'Information; PMSI), which provides medical information about all patients admitted to hospital in France, including discharge diagnoses encoded according to ICD-10, medical procedures and French diagnosis-related groups [12].

Approval from the French data protection agency (Commission Nationale de l'Informatique et des Libertés; CNIL) was obtained to conduct the present study.

### Study population and follow-up

All patients aged 40 to 79 years on 31 December 2006 and registered in the French national health insurance general scheme were eligible for inclusion. Included patients had to have filled at least one prescription for a glucose-lowering drug (metformin, sulfonylurea, pioglitazone, rosiglitazone, other oral hypoglycaemic agent and/or insulin) in 2006. The date of the first prescription of a glucose-lowering drug in 2006 constituted the study entry date.

Patients were excluded if they had bladder cancer diagnosed before study entry or within the first 6 months after study entry (screening for all PMSI discharge diagnoses since 2005 and all SNIIRAM LTDs since 1987). Patients with bladder cancer as a recognised occupational disease according to SNIIRAM were also excluded.

Follow-up started 6 months after study entry to allow for sufficient time to observe drug exposure. Follow-up ended at the time of the earliest of the following events: more than 4 consecutive months without having filled *any* drug prescription, diagnosis of bladder cancer, death from any cause or end of the observation period on 31 December 2009.

### Definition of exposure

Exposure to pioglitazone was determined from study entry onward and was defined as filling at least two prescriptions for pioglitazone over 6 consecutive months. Exposure was modelled as a time-dependent variable as follows: patients were considered not to be exposed from the time of study entry until the third month after meeting the above exposure conditions and exposed from the following month until the end of follow-up, even if the patient discontinued treatment. The same rule was applied with the other groups of glucose-lowering drugs.

The cumulative dose of pioglitazone was measured by determining the total dose dispensed since study entry. The cumulative dose was also considered to be a time-dependent variable by evenly distributing the total dose of each prescription over all days between the date of the prescription and the date of the subsequent prescription; the dose dispensed for the last prescription was not considered. The cumulative duration of pioglitazone use was calculated in the same way, counting 1 day for one pioglitazone tablet and 1 day for two tablets combining pioglitazone and metformin. Therefore, gaps in treatment did not contribute to cumulative duration. Cumulative doses and durations were classified according to the same intervals used in the KPNC study [6].

### Outcome

The main outcome was the incidence of bladder cancer during the follow-up period. Bladder cancer cases were identified by a hospital discharge diagnosis (ICD-10 code C67) and a specific surgical procedure (total cystectomy by laparotomy, or partial cystectomy by laparotomy or laparoscopy) and/or intravesical instillation of a pharmacological product by urethral catheter and/or chemotherapy and/or radiation therapy performed during the same hospital stay.

A broader definition of bladder cancer was used in an additional analysis, requiring only the presence of a discharge diagnosis of bladder cancer, including, in particular, endoscopic tumour resections for bladder cancer. The broader definition was also used to determine incidence rates of cancer in five other sites: lung (ICD-10 codes C33 and C34), head and neck (C00 to C14), colorectum (C18 to C21), female breast (C50) and kidney (C64).

### Confounding factors

The following potential confounding factors were available from the databases: age, sex and use of other glucose-lowering drugs.

Because the databases did not provide data on the duration of diabetes, the duration of full reimbursement for diabetes was used as a proxy for disease duration (in the subgroup of patients entitled to full reimbursement for diabetes treatment).

Data on smoking status were also absent from the databases. Therefore, we calculated two variables: (1) the use of drugs specific for chronic obstructive pulmonary disease (COPD; defined by having filled prescriptions for tiotropium bromide on at least three different dates in 2006); and (2) the presence of a hospital discharge diagnosis related to tobacco use in 2006 (ICD-10 codes F17, Z71.6 and Z72.0). Because only a small proportion of tobacco users could be identified by these two variables, we did not use this factor for model adjustment but only to compare pioglitazone-exposed to non-exposed patients.

### Statistical analysis

We compared categorical and continuous variables between pioglitazone-exposed and non-exposed patients using the χ^2^ test and Wilcoxon test, respectively, and used the Mantel–Haenszel method for age and sex-adjusted comparisons of variables related to tobacco smoking.

We calculated crude incidence rates of bladder cancer for pioglitazone-exposed and non-exposed patients by dividing the respective number of cases by the overall follow-up attributed to exposure and non-exposure, respectively (i.e. follow-up before the start of exposure was considered non-exposure).

We estimated the adjusted HR for bladder cancer incidence with pioglitazone exposure using a Cox proportional hazard model with the covariates age at study entry (in 5-year intervals), sex and use of other glucose-lowering drugs. The adjusted HRs for increasing cumulative dose and duration with exposure compared with non-exposure were estimated in the same way. The timescale of the model was duration of follow-up measured in calendar months.

To assess the sensitivity of the estimated HR with respect to several possible models, we performed the following additional analyses:

1. Use of age as the timescale (in calendar months) as an alternative approach to adjust for age;
2. Exclusion of patients with a pioglitazone prescription in the first 6 months of 2006 (followed by another prescription within 6 months);
3. In the subgroup of patients with full reimbursement for diabetes treatment for at least 1 year at study entry, an additional adjustment for duration of full reimbursement (in intervals of 1–4, 5–9, 10–14 and ≥15 years);
4. Use of the broader definition of bladder cancer, based simply on hospital discharge diagnoses without the additional requirement for treatment information used to define the primary outcome.
  Further analyses addressed the question of whether a possible increased risk of bladder cancer with pioglitazone exposure was specific to pioglitazone:
5. Analysis of the dose–effect relationship for another glucose-lowering drug, namely metformin;
6. Analysis of risk of cancer in five other sites (identified by hospital discharge diagnoses).
  All analyses involved the use of SAS statistical software, version 9.1 (SAS Institute, Cary, NC, USA). *p* < 0.05 was considered statistically significant.

## Results

### Description of the cohort population

**Fig. 1:**
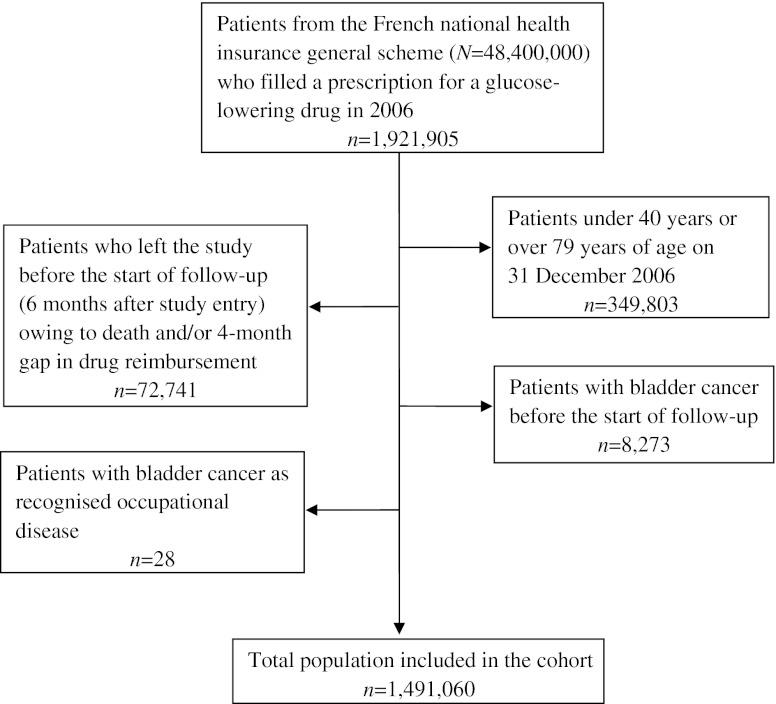
Flow chart of the pioglitazone and risk of bladder cancer study population

The study included 1,491,060 diabetic patients, 155,535 (10.4%) of whom were exposed to pioglitazone during the study period. Figure 1 shows a flow chart describing the study population.

The proportion of men was similar between pioglitazone-exposed and non-exposed patients: 53.8% vs 53.4% (Table 1). The mean age was lower for pioglitazone-exposed than non-exposed patients: 61.5 vs 63.4 years. Patients <70 years old represented 75.6% of exposed patients vs 67.1% of non-exposed patients. Compared with non-exposed patients, pioglitazone-exposed patients were more frequently exposed to metformin (82.7% vs 68.2%) and sulfonylureas (72.2% vs 55.5%) but less frequently to insulin (19.2% vs 27.6%). The proportion of patients with specific treatment for COPD was 0.89% for pioglitazone-exposed patients vs 1.11% for non-exposed patients. In addition, fewer pioglitazone-exposed than non-exposed patients had a discharge diagnosis related to tobacco smoking: 1.09% vs 1.41%, respectively. These differences were statistically significant, even after adjusting for age and sex.

**Table 1:** Demographics and glucose-lowering drug use by pioglitazone exposure: French cohort of diabetic patients aged 40–79 years (followed between 2006 and 2009)

Characteristic	Overall study population	Not exposed to pioglitazone		Exposed to pioglitazone	
		*n*	(%)	*n*	(%)
*N*	1,491,060	1,335,525		155,535	
Men	796,586	712,831	(53.4)	83,755	(53.8)
Age (years)					
40–44	55,903	49,789	(3.7)	6,114	(3.9)
45–49	94,472	82,593	(6.2)	11,879	(7.6)
50–54	158,419	137,813	(10.3)	20,606	(13.2)
55–59	237,091	207,912	(15.6)	29,179	(18.8)
60–64	237,327	210,837	(15.8)	26,490	(17.0)
65–69	230,578	207,344	(15.5)	23,234	(14.9)
70–74	254,631	232,172	(17.4)	22,459	(14.4)
75–79	222,639	207,065	(15.5)	15,574	(10.0)
Exposure to other glucose-lowering drugs					
Rosiglitazone	153,334	126,876	(9.5)	26,458	(17.0)
Metformin	1,039,844	911,143	(68.2)	128,701	(82.7)
Sulfonylurea	853,605	741,380	(55.5)	112,225	(72.2)
Other OHA	440,633	371,447	(27.8)	69,186	(44.5)
Insulin	398,835	368,913	(27.6)	29,922	(19.2)
Pioglitazone use during follow-up					
Cumulative dose (mg)					
<10,500	66,332	N/A		66,332	(42.6)
10,500–27,999	54,956	N/A		54,956	(35.3)
≥28,000	34,247	N/A		34,247	(22.0)
Duration of exposure (days)					
<360	58,756	N/A		58,756	(37.8)
360–719	36,482	N/A		36,482	(23.5)
≥720	60,297	N/A		60,297	(38.8)

Data are obtained from the SNIIRAM and PMSI databases

*p* < 0.01 for all comparisons

N/A, not applicable; OHA, oral hypoglycaemic agent

A total of 782,675 (52.5%) patients had received full reimbursement for diabetes treatment for at least 1 year. Within this patient subgroup, the duration of full reimbursement was shorter for pioglitazone-exposed than non-exposed patients (mean duration 6.5 vs 7.8 years). Patients who had received full reimbursement for at least 15 years represented 8.5% and 15.9% of patients in each group, respectively.

In the overall study population, the mean duration of follow-up (starting 6 months after study entry) was 37.5 months. For pioglitazone-exposed patients, the mean follow-up was 39.9 months, with 27.4 months attributed to exposure. The mean interval between the last and first pioglitazone prescription (from study entry onward) was 22.2 months. Among all pioglitazone-exposed patients, 38,925 (25.0%) filled a prescription for pioglitazone in January 2006 and 59,296 (38.1%) during the first 6 months of 2006.

### Association of pioglitazone exposure and bladder cancer

We identified 175 incident bladder cancers among pioglitazone-exposed patients compared with 1,841 incident bladder cancers among non-exposed patients. Crude incidence rates were 49.4 and 42.8 per 100,000 person-years, respectively. After adjusting for age, sex and use of other glucose-lowering drugs, pioglitazone exposure was significantly associated with incident bladder cancer (HR 1.22 [95% CI 1.05, 1.43]) (Table 2). There was no evidence of an association between exposure to other glucose-lowering drugs and incidence of bladder cancer. Analysis by sex revealed a significant association between pioglitazone and bladder cancer for men (adjusted HR 1.28 [95% CI 1.09, 1.51]) but not women (adjusted HR 0.78 [95% CI 0.44, 1.37]); we found only 13 cases of bladder cancer among pioglitazone-exposed women, compared with 213 cases among non-exposed women.

**Table 2:** Risk of bladder cancer with pioglitazone exposure: French cohort of diabetic patients aged 40–79 years (followed between 2006 and 2009)

Characteristic	Overall study population			Men			Women		
	(*N* = 1,491,060; 2,016 cases)			(*n* = 796,586; 1,790 cases)			(*n* = 694,474; 226 cases)		
	HR^a^	(95% CI)	*p* value	HR^a^	(95% CI)	*p* value	HR^a^	(95% CI)	*p* value
Sex, reference women									
Men	7.65	(6.66, 8.79)	<0.01		N/A			N/A	
Age (years), reference 40–44									
45–49	2.51	(0.85, 7.41)	0.10	2.39	(0.68, 8.40)	0.17	2.98	(0.35, 25.49)	0.32
50–54	5.70	(2.08, 15.60)	<0.01	6.64	(2.09, 21.13)	<0.01	2.90	(0.36, 23.15)	0.32
55–59	7.89	(2.93, 21.28)	<0.01	9.65	(3.08, 30.25)	<0.01	2.30	(0.29, 18.11)	0.43
60–64	15.34	(5.72, 41.13)	<0.01	18.82	(6.04, 58.67)	<0.01	4.31	(0.57, 32.37)	0.16
65–69	20.61	(7.70, 55.19)	<0.01	24.57	(7.89, 76.50)	<0.01	8.69	(1.19, 63.35)	0.03
70–74	30.37	(11.36, 81.17)	<0.01	35.54	(11.43, 110.49)	<0.01	14.74	(2.05, 105.93)	0.01
75–79	35.08	(13.12, 93.80)	<0.01	41.32	(13.28, 128.53)	<0.01	16.02	(2.23, 115.14)	0.01
Exposure to glucose-lowering drugs^b^									
Pioglitazone	1.22	(1.05, 1.43)	0.01	1.28	(1.09, 1.51)	<0.01	0.78	(0.44, 1.37)	0.39
Rosiglitazone	1.08	(0.92, 1.26)	0.35	1.10	(0.93, 1.30)	0.25	0.89	(0.53, 1.49)	0.66
Metformin	1.03	(0.93, 1.13)	0.60	1.03	(0.93, 1.14)	0.58	0.99	(0.75, 1.31)	0.96
Sulfonylurea	0.92	(0.84, 1.01)	0.08	0.91	(0.83, 1.01)	0.06	0.99	(0.76, 1.30)	0.95
Other OHA	1.00	(0.90, 1.11)	0.93	0.95	(0.85, 1.07)	0.40	1.36	(1.02, 1.81)	0.04
Insulin	1.08	(0.97, 1.21)	0.15	1.08	(0.96, 1.21)	0.20	1.10	(0.81, 1.50)	0.53

Data are from SNIIRAM and PMSI databases

^a^Adjusted HRs estimated from multivariate Cox model including age, sex (when applicable) and exposure to glucose-lowering drugs

^b^For each class of glucose-lowering drug, non-exposure was the reference group for calculation of the HR associated with exposure

N/A, not applicable; OHA, oral hypoglycaemic agent

We observed a dose–effect relationship, with a significant increase in risk for cumulative duration of exposure 12 to 23 months (adjusted HR 1.34 [95% CI 1.02, 1.75]) and ≥24 months (adjusted HR 1.36 [95% CI 1.04, 1.79]) (Table 3). The risk was increased 75% for cumulative doses ≥28,000 mg (adjusted HR 1.75 [95% CI 1.22, 2.50]). Somewhat larger estimates were observed for men for durations ≥24 months (adjusted HR 1.44 [95% CI 1.09, 1.91]) and cumulative doses ≥ 28,000 mg (adjusted HR 1.88 [95% CI 1.30, 2.71]).

**Table 3:** Risk of bladder cancer with increasing level of pioglitazone use during follow-up: French cohort of diabetic patients aged 40–79 years (followed between 2006 and 2009)

Exposure	Overall study population		Men		Women	
	HR^a^ (95% CI)	*p* value	HR^a^ (95% CI)	*p* value	HR^a^ (95% CI)	*p* value
Cumulative dose (mg)^b^						
<10,500	1.12 (0.89, 1.40)	0.34	1.17 (0.92, 1.48)	0.21	0.77 (0.36, 1.65)	0.51
10,500–27,999	1.20 (0.93, 1.53)	0.16	1.24 (0.96, 1.60)	0.10	0.84 (0.35, 2.06)	0.71
≥28,000	1.75 (1.22, 2.50)	<0.01	1.88 (1.30, 2.71)	<0.01	0.57 (0.08, 4.11)	0.58
Duration of exposure (days)^b^						
<360	1.05 (0.82, 1.36)	0.68	1.10 (0.84, 1.43)	0.49	0.76 (0.34, 1.72)	0.51
360–719	1.34 (1.02, 1.75)	0.03	1.39 (1.06, 1.84)	0.02	0.87 (0.32, 2.35)	0.79
≥720	1.36 (1.04, 1.79)	0.02	1.44 (1.09, 1.91)	0.01	0.71 (0.22, 2.23)	0.56

Data are from SNIIRAM and PMSI databases

^a^Adjusted HRs estimated from multivariate Cox model including age, sex (when applicable), level of pioglitazone use (i.e. cumulative dose and duration of exposure, respectively) and exposure to other glucose-lowering drugs

^b^Non-exposure was the reference group for calculating the HR associated with increasing level of pioglitazone use

The results from the additional analyses were as follows:

1. When age was used as the time scale, the adjusted HR was 1.28 (95% CI 1.10, 1.50);
2. After excluding patients with a pioglitazone prescription in the first 6 months of 2006, the adjusted HR was 1.17 (95% CI 0.94, 1.47, *p* = 0.17) and the adjusted HR for doses ≥28,000 mg was 1.94 (95% CI 0.73, 5.19, *p* = 0.19);
3. With an additional adjustment for duration of full reimbursement for diabetes treatment in the patient subgroup concerned, the adjusted HR was 1.20 (95% CI 0.98, 1.48). The duration of full reimbursement was not significantly associated with bladder cancer incidence: compared with a duration of 1–4 years, for 5–9 years, the adjusted HR was 0.98 (95% CI 0.84, 1.13); for 10–14 years, adjusted HR 0.94 (95% CI 0.78, 1.13); and for ≥15 years, adjusted HR 0.98 (95% CI 0.81, 1.18);
4. Analysis using the broader definition of bladder cancer also revealed an association with pioglitazone exposure (adjusted HR 1.13 [95% CI 1.03, 1.25]) and a dose–effect relationship for exposure duration ≥24 months (adjusted HR 1.23 [95% CI 1.03, 1.47]) and cumulative doses ≥28,000 mg (adjusted HR 1.44 [95% CI 1.13, 1.84]);
5. After excluding pioglitazone-exposed patients, we observed no dose–effect relationship between metformin use and bladder cancer: compared with non-exposure, the adjusted HRs for the dose quintiles (interval limits 870, 1,740, 2,461, and 3,185 g) were 1.00 (95% CI 0.88, 1.15), 1.14 (95% CI 1.00, 1.29), 1.05 (95% CI 0.90, 1.24), 1.07 (95% CI 0.87, 1.32) and 1.11 (95% CI 0.83, 1.49), respectively;
6. We observed no significant association between pioglitazone exposure and incidence of lung cancer, colorectal cancer, breast cancer in women, or kidney cancer (Table 4). For head and neck cancer, the adjusted HR was 0.85 [95% CI 0.73, 0.99]; *p* = 0.041.

**Table 4:** Risk of selected cancers with pioglitazone exposure: French cohort of diabetic patients aged 40–79 years (followed between 2006 and 2009)

Characteristic	Colorectal cancer			Female breast cancer			Kidney cancer			Lung cancer			Head and neck cancer		
	(*n* = 1,485,146; 10,618 cases)			(*n* = 671,510; 6,820 cases)			(*n* = 1,495,787; 2,861 cases)			(*n* = 1,493,472; 9,298 cases)			(*n* = 1,495,411; 2,868 cases)		
	HR^a^	(95% CI)	*p* value	HR^a^	(95% CI)	*p* value	HR^a^	(95% CI)	*p* value	HR^a^	(95% CI)	*p* value	HR^a^	(95% CI)	*p* value
Sex, reference women															
Men	1.71	(1.65, 1.78)	<0.01		N/A		1.89	(1.74, 2.04)	<0.01	4.78	(4.52, 5.05)	<0.01	4.12	(3.74, 4.54)	<0.01
Age (years), reference 40–44															
45–49	2.04	(1.40, 2.97)	<0.01	1.50	(1.20, 1.87)	<0.01	0.99	(0.66, 1.47)	0.95	2.53	(1.84, 3.47)	<0.01	2.51	(1.70, 3.71)	<0.01
50–54	3.93	(2.79, 5.55)	<0.01	1.61	(1.31, 1.99)	<0.01	1.60	(1.13, 2.27)	0.01	3.75	(2.79, 5.06)	<0.01	3.21	(2.22, 4.66)	<0.01
55–59	6.07	(4.33, 8.51)	<0.01	2.09	(1.72, 2.55)	<0.01	1.88	(1.34, 2.63)	<0.01	5.64	(4.21, 7.54)	<0.01	3.49	(2.43, 5.03)	<0.01
60–64	9.56	(6.84, 13.36)	<0.01	2.67	(2.20, 3.25)	<0.01	2.71	(1.95, 3.78)	<0.01	7.30	(5.46, 9.75)	<0.01	3.81	(2.65, 5.47)	<0.01
65–69	12.52	(8.96, 17.49)	<0.01	2.87	(2.36, 3.49)	<0.01	3.00	(2.16, 4.17)	<0.01	8.27	(6.19, 11.04)	<0.01	3.72	(2.59, 5.35)	<0.01
70–74	16.30	(11.68, 22.75)	<0.01	2.82	(2.32, 3.42)	<0.01	3.53	(2.55, 4.90)	<0.01	9.59	(7.18, 12.80)	<0.01	3.69	(2.57, 5.31)	<0.01
75–79	19.32	(13.84, 26.97)	<0.01	2.46	(2.02, 2.99)	<0.01	3.63	(2.61, 5.05)	<0.01	10.25	(7.68, 13.69)	<0.01	3.28	(2.28, 4.74)	<0.01
Exposure to glucose-lowering drugs^b^															
Pioglitazone	0.97	(0.90, 1.05)	0.45	0.91	(0.83, 1.00)	0.05	0.91	(0.79, 1.06)	0.22	0.94	(0.87, 1.02)	0.15	0.85	(0.73, 0.99)	0.04
Rosiglitazone	0.88	(0.82, 0.95)	<0.01	0.80	(0.73, 0.88)	<0.01	0.98	(0.86, 1.13)	0.80	0.91	(0.84, 0.99)	0.02	0.79	(0.67, 0.92)	<0.01
Metformin	1.02	(0.98, 1.07)	0.25	0.92	(0.88, 0.97)	<0.01	0.97	(0.89, 1.05)	0.39	0.88	(0.84, 0.92)	<0.01	0.75	(0.69, 0.81)	<0.01
Sulfonylurea	1.04	(1.00, 1.08)	0.05	0.97	(0.92, 1.02)	0.18	1.07	(0.99, 1.15)	0.09	0.93	(0.90, 0.97)	<0.01	0.89	(0.82, 0.96)	<0.01
Other OHA	1.03	(0.98, 1.08)	0.25	0.94	(0.89, 1.00)	0.05	1.05	(0.96, 1.15)	0.28	1.01	(0.96, 1.06)	0.62	0.88	(0.80, 0.97)	0.01
Insulin	1.05	(1.01, 1.11)	0.03	0.86	(0.81, 0.91)	<0.01	1.09	(1.00, 1.20)	0.05	1.23	(1.17, 1.29)	<0.01	1.24	(1.14, 1.36)	<0.01

Data are from SNIIRAM and PMSI databases

^a^Adjusted HRs estimated from multivariate Cox model adjusted for age, sex (when applicable), and exposure to glucose-lowering drugs

^b^For each class of glucose-lowering drug, non-exposure was the reference group for calculating the HR associated with exposure

N/A, not applicable; OHA, oral hypoglycaemic agent

## Discussion

We conducted this cohort study at the request of the AFSSAPS in response to a pharmacovigilance signal and the results of a US epidemiological study based on the KPNC database [6], suggesting a possible relationship between prolonged exposure (>2 years) to pioglitazone and increased risk of bladder cancer. Analysis of this cohort of 1.5 million diabetic patients, followed between 2006 and 2009, with median duration of pioglitazone exposure 1.5 years, showed that pioglitazone exposure was significantly associated with increased risk of bladder cancer (adjusted HR 1.22 [95% CI 1.05, 1.43]). Higher HRs were observed for high cumulative doses of pioglitazone (≥28,000 mg, adjusted HR 1.75 [95% CI 1.22, 2.50]) and long duration of exposure (≥24 months, adjusted HR 1.36 [95% CI 1.04, 1.79]). Sensitivity analyses of various models showed the robustness of the results.

These results confirm those of the KPNC study [6], based on 193,099 patients, including 30,173 exposed to pioglitazone with a median duration of exposure of 2.0 years: adjusted HR 1.2 [95% CI 0.9, 1.5]. The KPNC study revealed a comparable dose–effect relationship for exposure duration ≥24 months: adjusted HR 1.4 [95% CI 1.03, 2.0]. The similarity of the results between these two studies, conducted in different countries with distinct health systems and data-collection procedures, is striking and provides support for a causal association. In July 2011, the European Medicines Agency Committee for Medicinal Products for Human Use (CHMP) reported the results of a meta-analysis of randomised controlled clinical studies: 19 of 12,506 patients receiving pioglitazone had bladder cancer (0.15%) as compared with 7 of 10,212 patients not receiving pioglitazone (0.07%) [13].

One of the strengths of our study is that it used two comprehensive databases that were independent in terms of data collection. Data for medicinal product reimbursement are regularly and exhaustively submitted by all French pharmacists to the national health insurance network by electronic data interchange, and all French hospitals regularly submit their discharge data to the agency for information on hospital care (Agence Technique de l'Information sur l'Hospitalisation; ATIH) for planning and funding purposes. The a posteriori linkage between these two databases should have prevented observation bias, because identification of bladder cancer incidence is completely independent of measurement of pioglitazone exposure, in that this drug is mainly prescribed out of hospital.

Another strong point of the study is the systematic availability of data for all drugs eligible for reimbursement. The data on glucose-lowering drug use should therefore be comprehensive, because no self-prescribed medication for diabetes has marketing authorisation, and all glucose-lowering products are reimbursed by the national health insurance.

We minimised the misclassification of non-exposed patients as exposed patients by requiring exposed patients to meet the criterion of two pioglitazone prescriptions filled over a 6-month period. Patients who filled only one pioglitazone prescription (*n* = 15,756) were not classified as exposed. Some of these patients may have stopped treatment (e.g. because of an early adverse event) or never used the dispensed medication (e.g. because of a change in the initial treatment plan). Patients who received several pioglitazone prescriptions but never within a single 6-month period (*n* = 4,746) were also not classified as exposed. Some of these patients may have been actually exposed to pioglitazone. However, this classification error is unlikely to be substantial in view of the low use level, which is unlikely to modify the cancer risk. Furthermore, because these individuals represent a small proportion (3.1%) of those who met the exposure conditions and a very small proportion (0.4%) of the population classified as non-exposed, the potential impact on the estimate of the association is limited.

Two other elements support the specificity of the association of pioglitazone and bladder cancer: first, we found that use of none of the other oral hypoglycaemic treatments was associated with increased risk of bladder cancer, and second, pioglitazone exposure was not associated with increased risk of cancer in other sites. Insulin use appeared to be linked to increased cancer risk for all cancer sites studied (except for female breast cancer). This observation has been previously reported [14–17]. However, because our study was specifically designed to measure the risk of bladder cancer directly related to pioglitazone exposure, the results concerning use of other glucose-lowering therapies (oral or insulin) or other cancer sites should be interpreted with caution.

We observed a significant risk excess of bladder cancer in men only. Previous research has suggested that this effect in male rats can be prevented with dietary modification, suggesting a mechanism related to the acid milieu and urine bladder anatomy of male rats [18]. However, we believe that the power of our study is far too low to be sufficient to detect a risk for bladder cancer in women, with only 13 cases in exposed women vs 162 cases observed in exposed men.

Our study has several limitations. First of all, it lacks data on tobacco use, known to be the third main risk factor for bladder cancer after age and male sex [19, 20]. Nevertheless, several elements seem to address this limitation. First, the results reported by Lewis et al [6] for KPNC were similar after adjusting for age, sex and additional covariates, particularly smoking. Second, patients using specific drugs for COPD and with a discharge diagnosis related to tobacco were relatively few in the pioglitazone-exposed group, which suggests a lower proportion of tobacco use in exposed patients. Finally, the low risk of lung cancer and head and neck cancer with pioglitazone exposure also suggests a lower exposure to tobacco among pioglitazone users than among non-users. Therefore, the lack of adjustment for smoking would be expected to result, if anything, in an underestimate of the association between pioglitazone exposure and bladder cancer.

In addition, we do not report data on the duration of diabetes. In an additional analysis of patients entitled to full reimbursement for diabetes treatment, we used duration of full reimbursement as a proxy. Additional adjustment for this variable resulted in a similar HR for pioglitazone exposure and the duration of full reimbursement for diabetes treatment was not significantly associated with bladder cancer.

Although our report does not contain information on histological diagnosis, the definition of bladder cancer patients as those requiring relatively aggressive treatment should have minimised misclassification of non-cases as cases. However, bladder cancer patients not treated aggressively would have been missed. The incidence rates of bladder cancer we observed were similar to those reported by French cancer registries [21] up to the age of 80 years, for both men and women. However, several studies of diabetic populations have reported a slight increase in cancer risk in general [17] and bladder cancer risk in particular. The meta-analysis by Larsson et al reported an increased risk of bladder cancer (relative risk 1.24 [95% CI 1.08, 1.42]) when comparing type 2 diabetic individuals with non-diabetic individuals [22]. French cancer registries are population-based and these findings suggest a slight underestimation of the number of bladder cancer cases in our study. However, with our data-collection method (hospital discharge diagnoses and specific therapy), this underestimation concerns both pioglitazone-exposed and non-exposed patients. This non-differential misclassification of disease would be expected to result in an underestimate of the risk associated with exposure to pioglitazone. Furthermore, our analysis using a broader definition of bladder cancer, testing only hospital discharge diagnoses, also indicates a significant association between pioglitazone exposure and bladder cancer risk.

We observed a dose–effect relationship for pioglitazone exposure only. If the increased incidence of bladder cancer was due to deterioration of diabetes and not directly linked to pioglitazone use, we would expect a similar increased risk with the highest doses of metformin. Our findings, therefore, support the theory that the increased bladder cancer risk among patients exposed to pioglitazone cannot be explained by disease progression.

Information on drug exposure before 2006 was not available. About one-third of pioglitazone-exposed patients filled a prescription for pioglitazone during the first 3 months of 2006, but pioglitazone use in France was less frequent before than after 2005 (the number of packages reimbursed in 2004 represented approximately 20% of those reimbursed in 2006). Pre-study exposure to pioglitazone would therefore have concerned a maximum of one-third of exposed patients and the duration of pre-study exposure would rarely have exceeded 1 year. Moreover, the HR estimates of our additional analysis in new users of pioglitazone were in line with those of the main analysis, but the confidence intervals were broader as a result of the smaller number of patients.

The French SNIIRAM and PMSI databases have been extensively used in pharmacoepidemiological studies. A search on 1 June 2009 for published studies involving drug reimbursement data from SNIIRAM found 110 articles [23]. The combined use of SNIIRAM and PMSI databases in observational pharmacoepidemiological studies is a promising approach to assess the potential for use of a drug to produce serious adverse reactions leading to hospital admission.

In summary, in this cohort of 1.5 million diabetic patients followed between 2006 and 2009, pioglitazone exposure was significantly associated with increased risk of bladder cancer. Risk estimates were similar to those observed in the KPNC cohort [6] but in a much larger population and in France. Despite its limitations, this study, using linked data from the SNIIRAM health insurance information system and the PMSI database in France, demonstrated that these medico-administrative databases can be helpful in addressing a large number of public health issues such as estimation of disease frequencies and related healthcare spending, as well as assessment of drug safety.

## Abbreviations

AFSSAPS: Agence Française de Sécurité Sanitaire des Produits de Santé (French medicines agency)
COPD: Chronic obstructive pulmonary disease
ICD: International Classification of Diseases
KPNC: Kaiser Permanente Northern California
LTD: Long-term disease
PMSI: Programme de Médicalisation des Systèmes d'Information (French hospital discharge database)
PPAR: Peroxisome proliferator-activated receptor
SNIIRAM: Système National d'Information Inter-régimes de l'Assurance Maladie (French national health insurance information system)
